# miRGalaxy: Galaxy-Based Framework for Interactive Analysis of microRNA and isomiR Sequencing Data

**DOI:** 10.3390/cancers13225663

**Published:** 2021-11-12

**Authors:** Ilias Glogovitis, Galina Yahubyan, Thomas Würdinger, Danijela Koppers-Lalic, Vesselin Baev

**Affiliations:** 1 Faculty of Biology, University of Plovdiv, Tzar Assen 24, 4000 Plovdiv, Bulgaria; ilias@uni-plovdiv.bg (I.G.); gyahubyan@uni-plovdiv.bg (G.Y.); 2 Department of Neurosurgery, Cancer Center Amsterdam, Amsterdam University Medical Centers, VU University Medical Center, De Boelelaan 1117, 1081 HV Amsterdam, The Netherlands; t.wurdinger@amsterdamumc.nl (T.W.); d.lalic@amsterdamumc.nl (D.K.-L.)

**Keywords:** miRNAs, isomiRs, NGS, Galaxy, bioinformatics tools

## Abstract

**Simple Summary:**

MicroRNAs are essential regulators of gene expression and potential non-invasive biomarker candidates for various human cancers as they can be detected in bodily fluids. Several tools have been developed to analyze small RNA-sequencing data; however, they have limitations and restrictions such as lack of optimal configuration, parameterization, and interoperability with other tools and platforms. miRGalaxy is an open-source, Galaxy-based framework for analyzing NGS data focusing on microRNAs and their sequence variants—isomiRs. Galaxy is a web-based platform for data-intensive biomedical research, allowing user-friendly analysis and accessibility to hundreds of tools. miRGalaxy is designed specifically for identifying and classifying human microRNAs and isomiRs, as well as detecting deregulated microRNAs and isomiRs between two test groups, summarized by output visualization. By examining the differential expression of individual isomiR species across samples, miRGalaxy can help discover novel biomarkers.

**Abstract:**

Tools for microRNA (miR) sequencing data analyses are broadly used in biomedical research. However, the complexity of computational approaches still remains a challenge for biologists with scarce experience in data analytics and bioinformatics. Here, we present miRGalaxy, a Galaxy-based framework for comprehensive analysis of miRs and their sequence variants—miR isoforms (isomiRs). Though isomiRs are commonly reported in deep-sequencing experiments, their detailed structure complexity and specific differential expression (DE) remain not fully examined by the majority of the available analysis tools. miRGalaxy encompasses biologist-user-friendly tools and workflows dedicated to the analysis of the isomiR-ome and its complex behavior in various biological samples. miRGalaxy is developed as a modular, accessible, redistributable, shareable, and user-friendly framework for scientists working with small RNA (sRNA)-seq data. Due to its modular workflow, advanced users can customize the steps and tools for their needs. In addition, the framework provides an analysis report where the significant output results are summarized in charts and visualizations. miRGalaxy can be accessed via preconfigured Docker image flavor and a Toolshed installation if the user already has a running Galaxy instance. Over the last decade, studies on the expression of miRs and isomiRs in normal and deregulated tissues have led to the discovery of their potential as diagnostic biomarkers. The detection of miRs in biofluids further expanded the exploration of the miR repertoire as a source of liquid biopsy biomarkers. Here we show the miRGalaxy framework application for in-depth analysis of the sRNA-seq data from two different biofluids, milk and plasma, to identify, annotate, and discover specific differentially expressed miRs and isomiRs.

## 1. Introduction

The rapid development of high-throughput sequencing technologies and related bioinformatics tools for processing RNA sequencing data has led to the identification of a huge number of sequences having the distinctive features of miRs [1,2,3,4,5,6]. They are produced by different types of genomic loci (miR genes, non-miR genes, and others), the largest being the contribution of miR genes, further supported by a huge number of studies in various taxa [7,8,9]. Any such sequence could be called a miR as long as it has structural features of being short and single-stranded, and functional significance mediated by an Argonaute protein [6]. What distinguishes classically defined miR or “reference sequence miR” (RefSeq miR) from its sequence variants, called isoforms or isomiRs, is its superiority in being mapped to a miR gene before all other sequence variants that map to the same gene. In addition, the first mapped sequence fully aligned to the genomic (miR gene) sequence is often deposited in an appropriate database. Interestingly, the reference miR sequences annotated as the most dominant sequence derived from a particular miR gene are often found to be less represented (e.g., expressed) than its isomiR(s) [6].

Similar to RefSeq miRs, the expression profiles of isomiRs can vary during the individual development of a healthy organism, and their qualitative and quantitative profile can be greatly altered in diseased (pathological) tissues, reflecting the disease progression and drug treatment effects. IsomiR’s signature can distinguish tumor cells from normal; moreover, it can even identify different types of cancer and their subtypes [10,11]. Furthermore, the rapidly growing miR/isomiR repertoire in liquid biopsy assays makes miR/isomiR signatures a promising source for the development of new molecular biomarkers.

Novel sequencing technologies paired with various bioinformatics tools have led to accelerated technological progress in sRNA and miR analysis and exploration of their repertoire. Nevertheless, the blend of different bioinformatics applications remains essential to draw significant outcomes from the sRNA studies. Thus, modular and user-friendly graphic user interface (GUI) tools would greatly improve and promote such studies.

Almost all bioinformatics tools that analyze sRNA-seq data are implemented and performed using Linux/Unix servers or clusters. However, the analysis and the data output of the results generated by these operating systems are difficult for most biology scientists. There is a growing need to develop efficient applications having a user-friendly GUI and visualizations and thus to provide biologically understandable summarization of the results.

Galaxy [12] is an open, web-based platform for modular data-intensive biomedical research. It keeps track of history, and all analyses can be rerun, providing accessibility and reproducibility of the performed analysis. Furthermore, the Galaxy community is very active, and a great number of Linux-based bioinformatics tools are included in Galaxy tanks as a modular system providing great GUI for biologists. Authors are enabled to develop Galaxy-integrated tools that can be shared via the Galaxy toolshed, which serves as an app store, providing other users with the opportunity to install them to their Galaxy instances in an easy click-and-install way.

Recently, we have reviewed currently available computational tools for isomiRs analysis [13]. Most of the tools emphasize the isomiR identification but failed to extend the analysis further, exploring the DE of individual isomiR species across samples and thus providing the means for novel biomarker discovery.

In this context, we developed miRGalaxy, an open-source, Galaxy-based framework for miR and isomiR analysis from NGS data. It integrates several dedicated tools and workflows for in-depth isomiR analysis, including more than 100 default tools for NGS data analysis. miRGalaxy is developed as a modular, accessible, redistributable, shareable, and user-friendly framework for scientists working with miR data. This novel framework combines our specifically developed isomiR tools with already curated Galaxy modules, thus providing the user with the flexibility to utilize the framework as it is, or to modify it with other preferred tools (e.g., the default steps of quality check, adapter trimming, mapping, etc.). Such customized utilization ensures that the framework can be used by basic as well as advanced users working with miR data.

**Figure 1:**
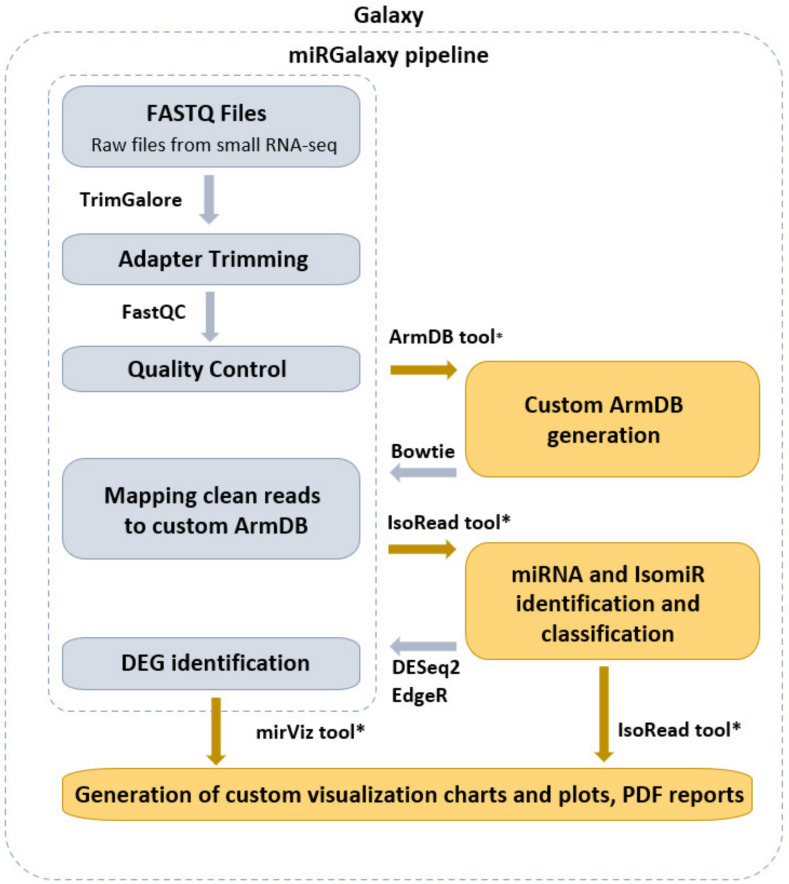
miRGalaxy workflow overview (the tools marked with an asterisk are newly developed for the miRGalaxy framework).

miRGalaxy tool suite is composed of three main tools—ArmDB, IsoRead, and miRViz (Figure 1). This allows the user to identify and discover miRs and their template and non-template isomiRs, to further process in-depth expression analysis of individual isomiRs across samples, and to generate a custom visualization report output of their study.

To enhance miRGalaxy accessibility, all tools, workflow, and their dependencies have been integrated into a Galaxy Docker flavor (~10 GB), providing easy access and hassle-free installation on a server or a local user PC machine.

## 2. Materials and Methods

miRGalaxy is a Galaxy-based framework integrating curated state-of-the-art NGS tools extended with three new tools dedicated to miR and isomiR identification, classification, and expression assessment. miRGalaxy is built on the top of the Galaxy Docker image (Galaxy version 20.09) as a flavor instance. The advanced miRGalaxy workflow is shown in Figure 1. The workflow comprises well-known steps for data quality control (QC), read rimming, mapping, and DE (shown in grey) supported by default Galaxy tools—FastQC (v0.72) [14], MultiQC (v1.9) [15], TrimGalore (v0.6.3) [16], Bowtie (v1.2.0) [17], and DESeq2 (v2.11.40.6) [18]/EdgeR (v3.24.1) [19,20], respectively. Data processing intended to identify RefSeq miRs and template/non-template isomiRs is executed by two new tools—ArmDB and IsoRead. As a final step of the workflow, another new dedicated tool—miRViz, provides users with PDF reports, in which outputs are collected and visualized with custom charts. The newly developed Galaxy tools were written in Python 3.7.4. The following libraries—fpdf (v1.7) [21], numpy 1.17.3 [22], pandas 1.0.3 [23], matplotlib 3.1.2 [24], and logomaker 0.8 [25] were used for tool implementation and graph rendering.

### 2.1. Pre-Processing Steps

Users are required to have sRNA-seq data as FASTQ files to run the miRGalaxy workflow. The user can upload FASTQ files and can organize sample collections in groups. First, the quality of sRNA-seq data is examined using FastQC with default parameters, followed by adapter trimming using TrimGalore (with an option to detect the adapters automatically).

### 2.2. ArmDB Tool

**Figure 2:**
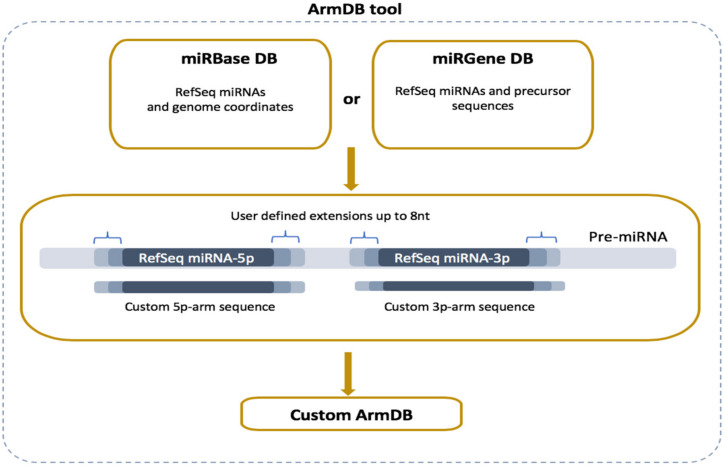
Overview of ArmDB tool. The tool can use data from miRBase or miRGene DBs to generate a custom database with 5′- and 3′-arm sequences used as a reference for read mapping in downstream analysis. The arms can be generated with user-defined nucleotides extension of the RefSeq sequences (up to 8 nt).

Read alignment is preceded by generating a custom reference database with the two precursor arms (5p and 3p) of the RefSeq miRs executed by the newly developed ArmDB tool (Figure 2). Each custom miR arm is a fragment of a published stem-loop miR precursor (pre-miR) and comprises the corresponding RefSeq miR sequence extended by several nucleotides (user-defined) at the 5′- and 3′-ends. To do that, all RefSeq miRs and pre-miR sequences are parsed from miRBase or miRGene DBs according to user preferences [26,27]. More specifically, if a user chooses miRGene DB, the ArmDB tool extends the RefSeq miR sequence (3p and/or 5p) with several nucleotides (maximum 8 nt) on both ends in agreement with the precursor, and the generated sequence is saved as a custom arm (Figure 2). If a user prefers miRBase, ArmDB downloads the genome coordinates of RefSeq miRs as a GFF3 file. Then, the genome coordinates are modified to have additional nucleotides, and the custom arm is extracted from the user-defined genome with GetFasta tool from BEDtools (v2.28.0) suite [28]. We consolidate the information of the Galaxy-integrated genomes with the miR DBs versioning and provide a selection of 49 ready-to-use reference genomes. As output files, the ArmDB tool returns FASTA files with the generated custom miR arms (and some additional files: FASTA files with RefSeq miRs and pre-miRs if miRGene was selected, or GFF3 files with the miR genome coordinates if miRBase was selected).

### 2.3. Read Mapping

For mapping, miRGalaxy workflow uses Bowtie which receives sRNA-seq data and the custom arm DB as a reference from ArmDB tool [17]. Alignment mode is set to zero mismatches, and all valid alignments per read are reported. SAM file containing all mapped and unmapped reads is set as output.

### 2.4. IsoRead Tool

**Figure 3:**
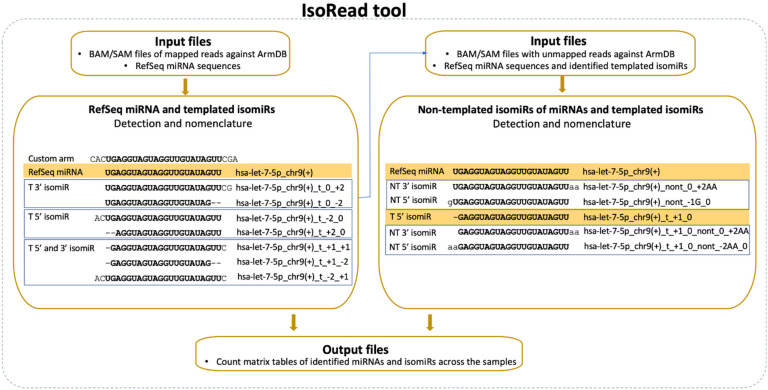
IsoRead tool overview. The tool performs scanning of the input SAM files from the read mapping. It discovers and classifies miRs and their isoforms in two main types—templated isomiRs (_t) and non-templated isomiRs (_nont) across samples. The coordinates in the name extension show the sequence offset relative to the RefSeq sequence. The IsoRead tool generates output files as count matrices with all discovered miRs and isomiRs.

The second newly developed tool—IsoRead is aimed at identifying and classifying RefSeq miRs and isomiRs in sequencing reads (Figure 3). The main isomiR types incorporated in the algorithm are: (i) template isomiRs whose ends are offset from the corresponding ends of the RefSeq miR-5′-shifted with one or more nt (5′-isomiRs), 3′-shifted with one or more nt (3′-isomiRs), 5′- and 3′-shifted with one or more nt (5′3′-isomiRs); and (ii) non-template isomiRs (non-template additions at the 3′-end of the RefSeq miR up to 3 nt).

The SAM files from the alignment step and FASTA files with RefSeq miR sequences from miRBase or miRGene are set as input. The tool handles SAM files as groups (Control-Treated, Wildtype-Mutant, Healthy-Cancer, e.g.,) and keeps only the sequences with a length between 18 and 26 nt for each file. ArmDB-mapped and unmapped reads are classified according to the following hierarchical scheme. First, IsoRead performs an identity check of all mapped reads by comparing with the selected miR DB, and reads are assigned to RefSeq miRs if they have perfect matching. Second, the different types of template isomiRs are determined from the other mapped reads in the SAM file, depending on whether they contain a partial or extended RefSeq miRs sequence. Their names are composed of the name of the RefSeq miR plus coordinate numbers showing the offset of the end of isomiR according to the ends of the reference sequence (Figure 3). Third, the user can also choose to continue with detecting the non-template isomiRs. In this case, non-template isomiRs are identified from the unmapped pool of reads in the SAM file. IsoRead combines the RefSeq miR and discovered template isomiR sequences from the previous step and uses them as reference sequences for the non-template isomiR identification. In that way, we can discover non-template isomiRs of the already discovered RefSeq miRs and the template isomiRs. The nomenclature procedure is the same as described above, the name being extended to show the number of additional nt and their corresponding coordinates. If non-template isomiR of a template isomiR is discovered, its name carries information about both the shifting of the template sequence relative to the RefSeq miR and the number of non-template nt additions. If a detected sequence is matched to more than one RefSeq miR sequence, it keeps all records by collapsing them in one entry by merging their names to solve the problem with duplications. As output, the tool produces tabular files with all the detected miRs and isomiRs per category for every SAM file as a database for the user and two types of count matrices containing the copy number of each entry. One of the count matrix types is compatible with the DESeq2 and EdgeR tools so that the user can continue with DE analysis. The other types of count matrices generated per miR category (RefSeq miRs, template isomiRs, and non-template isomiRs) and per group are enriched with read sequences. Apart from the tabular files, the tool produces a PDF report file with several custom graphs and statistical information about the isomiR profiles in the user data.

### 2.5. DE Step

The statistical step and identification of DE miRs and isomiRs can be performed either by DESeq2 or EdgeR tools which users can choose (we provided dedicated workflow with each tool). In this step, the generated matrix tables with all identified miRs and isomiRs (template or non-template), their copy numbers across all samples, and groups are fed into the desired statistical module. We have included an extra filtering step in our workflow, where the user can choose a parameter to filter the output DE miRs and isomiRs by mean read number, log2FC, *p*-value, and/or *p*-adjustment. As default parameters, we have set the final filtering of DE table by *p*-adj < 0.05 and |Log2FC| > 1.

### 2.6. miRViz Tool

The tool is an additional and optional follow-up to the DE analysis, rendering more custom graphs from the DE results. It generates graphs with the top DE miRs (the number can be specified by the user in the options range of 10 to 50) and miR and isomiR profiles by arm. Top miR and isomiR selection are achieved by a combination of criteria according to the user preferences. These criteria are mean read number (only available for DESeq2 outputs), log2FC, *p*-value, and/or *p*-adjustment. The tool receives DE results from the DESeq2 or EdgeR tools as input files and produces a pdf report.

### 2.7. Availability of the Framework, Installation, and Running miRGalaxy

Running the containerized miRGalaxy is easy and requires the user to install Docker [29] and to start the miRGalaxy image with the following command:

$ docker run −d − p 8080:80 glogobyte/mirgalaxy

As Galaxy, miRGalaxy is production-ready, pre-configured, and preinstalled with all required tools needed for the analysis. The image will be automatically downloaded and deployed for miRGalaxy with a ready-to-run analysis platform. Additionally, if the user already has local Galaxy and wants to use our workflow and tools within its own running instance, the user can install only the tools (ArmDB, IsoRead, and miRViz) from the Galaxy Toolshed [30] An archive of the Dockerfile and workflow files can be downloaded from the Github repository at https://github.com/Glogobyte/miRGalaxy.

## 3. Results and Discussion

### 3.1. miRGalaxy Outlines

We have developed miRGalaxy, an open-source, Galaxy-based framework for miR and isomiR analysis to address several essential needs in the field. First, miRGalaxy provides a modular workflow for miR/isomiR analysis to further expand the possibilities of detailed isomiR identification, classification, and expression assessment. The isomiR repertoire comprises all template and non-template variations of RefSeq miRs, extended with the non-template variations of the discovered isomiR species. Moreover, our workflow is able not only to perform genome-wide miR/isomiR profiling but also to assess the DE of each individual isomiR species across samples. Second, some steps of miRGalaxy can be highly customized as users can modify main parameters or even use their favorite tool instead of the default one integrated into our ready-to-use workflow. It allows great flexibility and sustainability over time, as the Galaxy tool development maintains high speed and new advanced tools additions. In this way, the user can explore the state-of-the-art tools for the general steps of the workflow such as pre-processing of raw files, adapter trimming, read mapping, statistics, etc.

**Figure 4:**
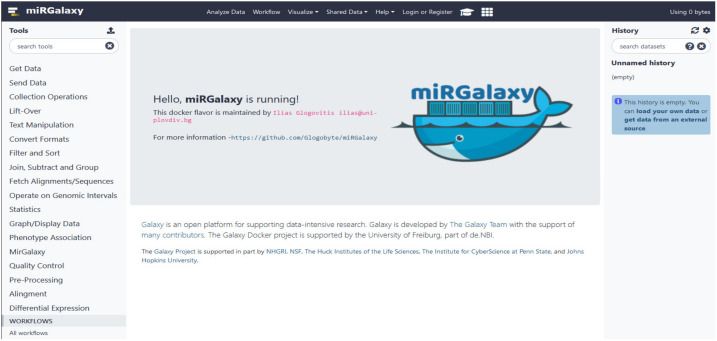
miRGalaxy GUI. The miRGalaxy workflow can be accessed via the top menu “Workflow”.

**Figure 5:**
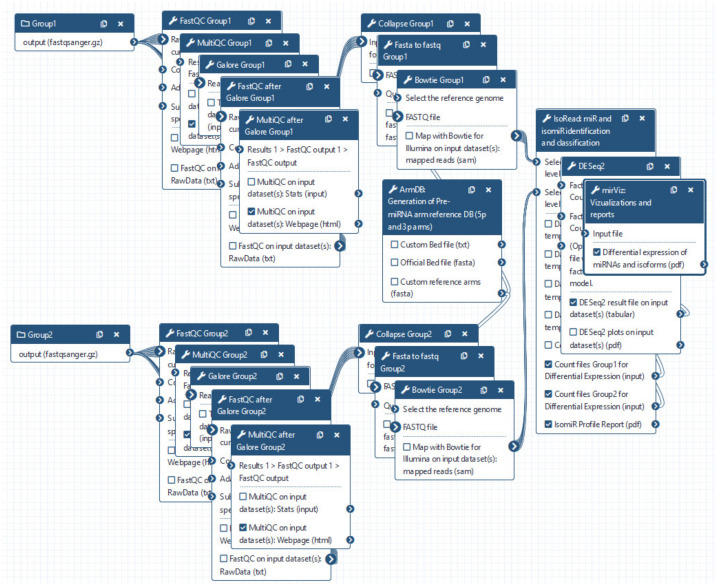
miRGalaxy workflow. User is asked to upload FASTQ files and run the workflow (default options are preset for every step of the analysis and can be changed upon user preferences).

We provide miRGalaxy as a Docker flavor image so that the user can have a ready-to-run platform with no need for pre-configuration. After the image run (see Materials and Methods), the user can choose to run the automatic miRGalaxy workflow located in the top menu “Workflow” or manually run the framework tools (Figure 4). Before starting the workflow, the user must upload FASTQ datasets and create collections for the data groups (e.g., defined as Group 1 and Group 2). The user is ready to run the workflow with the default preconfigured options as the pipeline requires only the input raw read files (Figure 5). QC and MultiQC reports before and after the adapter trimming are provided for each dataset group. At this point, the user can check if datasets have the expected sRNA length distribution profile and total clean read number resulting from the adapter removal. MultiQC will concatenate and summarize every FastQC report and give a summary for each of the groups. As the workflow is modular and customizable, advanced Galaxy users may choose to use other tools for QC, adapter trimming, and mapping, which makes the miRGalaxy pipeline flexible and robust.

### 3.2. miRGalaxy Implementation

Liquid biopsy is emerging as a minimally invasive sampling of body fluids for the purpose of disease diagnosis, monitoring therapeutic effectiveness, and prognosis [31,32]. Different biofluids such as blood, urine, saliva, milk, and cerebrospinal fluid (CSF) can be used as a source of liquid biopsy [33]. sRNA-seq studies using different sources of biofluids confirmed the detection of miRs and implicated their expression analysis as a useful approach towards identification of easily accessible molecular biomarkers. To test the miRGalaxy tool suite for its performance in the analysis of liquid-biopsy-derived sRNA-seq data, and for in-depth characterization of the miR/isomiR repertoires, we explored publicly available data sets from two different human biofluids, milk and plasma [34]. Additionally, we tested miRGalaxy tool performance by using the sRNA-seq data published under GEO repository ID accession: GSE160252 and obtained from profiling circulating blood platelets samples of asymptomatic individuals and cancer-diagnosed patients [35]. 

In our case study on milk and plasma samples, we used the raw sequencing reads and data (samples annotations) published under GEO repository ID accession: GSE107524 [34]. QC step of the sRNA-seq datasets was run on 15 raw data files (5 plasma samples defined as Group 1 and 10 milk samples defined as Group 2) to detect potential technical issues during data collection. We used the default options in the ArmDB tool—miRBase as a miR source database, *Homo sapiens* hg38 genome, and 6 nt extensions of the RefSeq miR sequences. The custom ArmDB database was used as a reference for the mapping tool (in our case Bowtie, but users can customize the workflow and use another mapping tool for short reads) to align group sample reads. From miRBase (v22), the ArmDB tool created 2883 custom arm sequences corresponding to the 5′- and 3′-arms of the RefSeq miRs.

**Figure 6:**
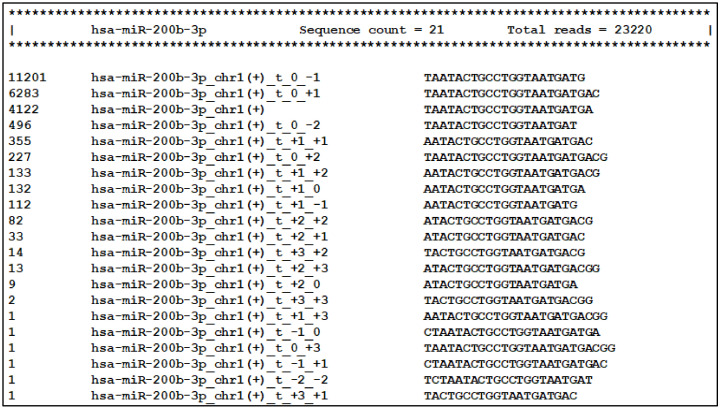
Visualization of the database comprising all reads produced by miR arms. Templated isomiR sequences of miR-200b-3p discovered in the milk sample are presented as an example.

Next, the main tool of the miRGalaxy workflow—IsoRead mined the SAM mapping files to discover and classify miRs and their template and non-template (optional) isomiRs in each sample of the groups. Here the tool created detailed count matrices with identified miRs and isomiRs in each sample. Furthermore, all detected sequences produced by miR arm were collected in a database. As an example, summarization of miR-200b-3p and its isomiRs was extracted from the database and is presented in Figure 6 (representing a snapshot of the screen display).

We were able to identify 503 and 903 RefSeq miRs, 2531 and 6675 template isomiRs, and 6207 and 23,832 non-template isomiRs in the plasma and milk samples, respectively. The five most abundant miRs and isomiRs in the analyzed samples from milk and plasma are shown in Table 1a and from circulating platelets samples in Table 1b. The complete data analysis of circulating platelets is provided in the supplementary files (Tables S4–S6, Figures S3 and S4).

**Figure 7:**
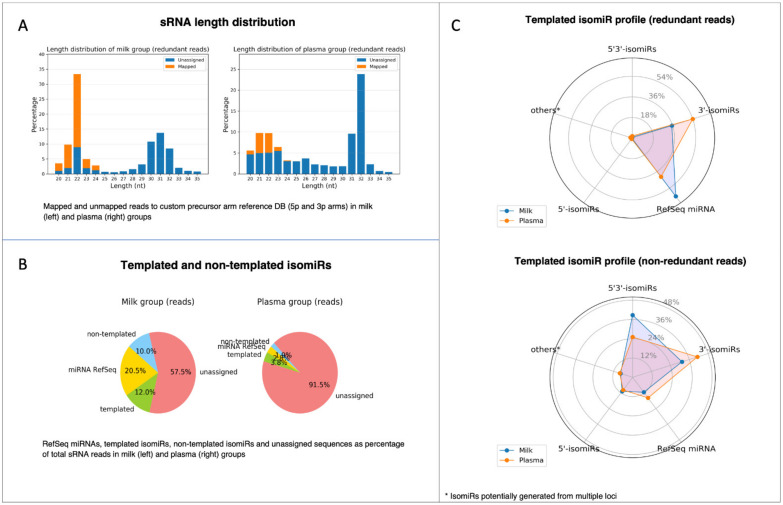
Visualization of isomiR profile PDF report produced by the miRViz tool. (**A**) Length distribution of sRNA reads by sample groups; (**B**) pie charts depict the percentage distribution of RefSeq miRs, templated and non-templated isomiRs; (**C**) spider diagrams with the distribution of different subtypes of the template isomiRs (3′-isomiRs, 5′-isomiRs, and 5′3′-isomiRs) in redundant and non-redundant datasets.

Before feeding the count matrices into the statistical tool for DE analysis, the IsoRead module outputs an isomiR profile report with several summarization charts for each of the analyzed dataset groups (Figure 7). The first chart provided in the isomiR profile report represents the length distribution of sRNA reads by sample groups (Figure 7A). Sequences of length between 20 nt and 35 nt are shown, and the percentage of mapped and unmapped reads is color coded. The length distribution profiles of the two analyzed groups exhibit a peak at 22 nt characteristic for miRs. In general, high peaks corresponding to the lengths of certain RNA species should be distinguishable: miRs should form a narrow peak around 20–22 nt. If there are no clear peaks, this may suggest an issue related to RNA quality and degradation. A pie chart depicts the percentage distribution of the identified RefSeq miRs, template and non-template isomiRs, and unassigned reads in each group (Figure 7B). These charts enable the user to obtain information about the relative quantities of miRs in each group; in our case, reads that have been assigned to miRs and isomiRs were 42.5% in the milk group and 8.5% in the plasma group. Furthermore, spider diagrams reflect the distribution of the different subtypes of the template isomiRs (3′-isomiRs, 5′-isomiRs, and 5′3′-isomiRs) in redundant and non-redundant datasets (Figure 7C), giving an idea of the sequence diversity in datasets.

At this step, the IsoRead tool provides detailed count matrices with all discovered miRs and isomiRs (including sequence and copy number data) in the samples. This output can be used for various statistical tools to produce DE analysis. We have integrated our workflow to use state-of-the-art tools such as DESeq2 and EdgeR, which are widely adopted in the miR DE analysis [18,20]. Nevertheless, our modular workflow allows advanced users to apply their favorite statistical tool.

Unlike other available isomiR instruments, miRGalaxy uses advanced nomenclature and sequence tracking across the samples. This further provides comprehensive data for each individual isomiR for the statistical tools to identify specific DE isomiRs within template and non-template subsets. miRGalaxy identifies not only non-template isomiRs of RefSeq miRs but also non-template isomiRs of the discovered template isomiRs. Due to this detailed profiling, the miR-ome landscape in the analyzed datasets is enriched and provides a larger amount of data for DE analysis. In our case, we have used DESeq2 as a DE tool to compare data from plasma and milk samples. The final DE output was filtered, with the default filtering using a cutoff of *p*-adj < 0.05 and |Log2FC| ≥ 1 (Figure S2).

**Figure 8:**
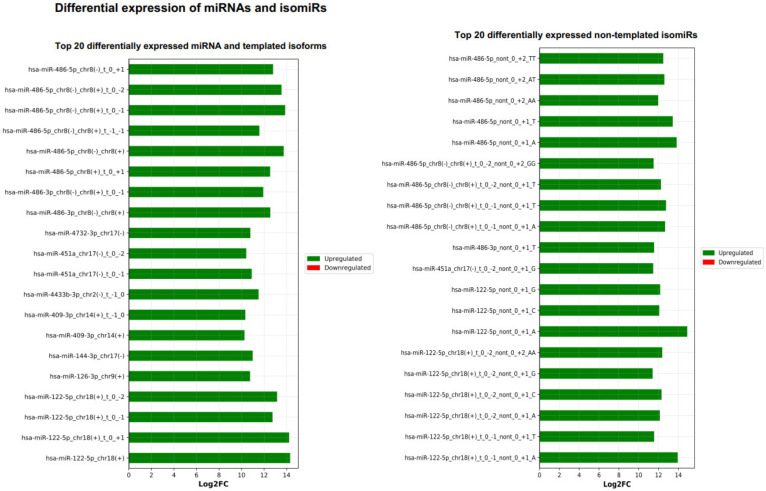
Top 20 differentially expressed miRs and isomiRs (template and non-template) generated by miRViz tool.

**Figure 9:**
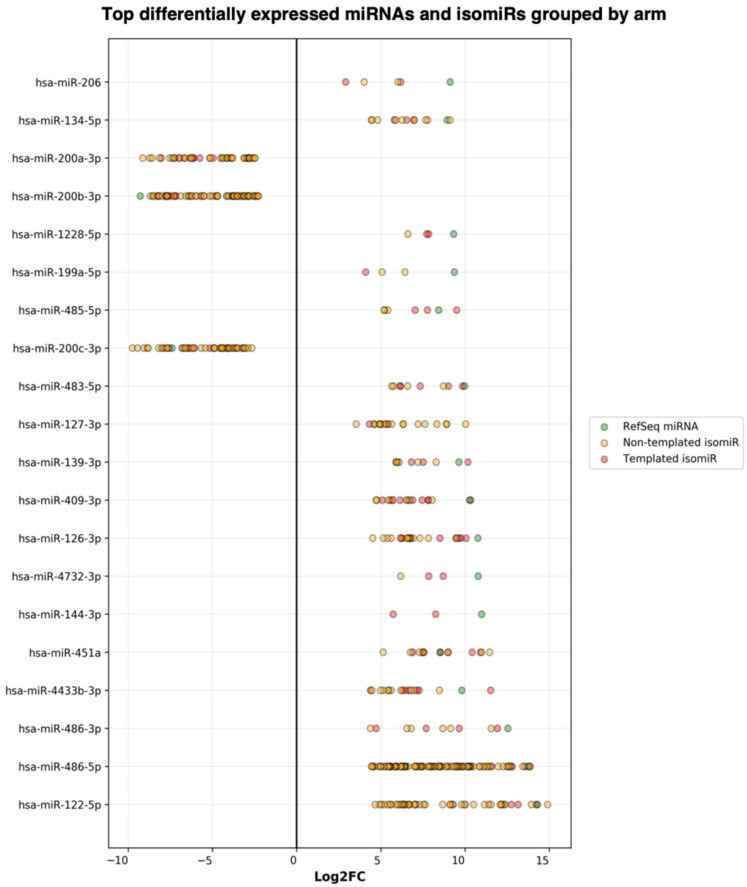
Summary of the top DE miRs and isomiRs grouped by miR arm. The number of miR arms presented in the report can be specified by the user.

Next, the DE result files were forwarded to our third tool—miRViz, which renders customized charts within a PDF report (Figure 8 and Figure 9). Users can set a cut-off and display the top differentially expressed entries with separate charts—one for RefSeq miRs and template isomiRs and another one for non-template isomiRs (optional in the workflow). Furthermore, we provide a summary chart of the miR arms with all assigned miR and isomiR species associated with log2FC. In our case datasets, we have identified a total of 3627 sequences DE in the plasma samples, of which 268 are RefSeq miRs, 1014 template isomiRs, and 2346 non-template isomiRs. With the help of the miRViz tool, we have generated a custom chart of the top 20 DE miRs and isomiRs grouped by arms (Figure 9). The most upregulated RefSeq miRs in plasma were miR-122-5p (log2FC 14.3) together with its isomiR miR-122-5p_t_0_+1 (log2FC 14.2) and the most downregulated RefSeq miRs were miR-200b-3p (log2FC −9.2) and miR-200a-5p (log2FC −7.4) and from isomiRs were miR-30a-3p_t_0_−2 (log2FC −8.5) and miR-200b-_3p_t_+1_+1 (log2FC −8.2). Recently, several studies observed that the circulating miR-122-5p was highly expressed in plasma [36,37,38] and miR-200a, miR-200b, and miR-30a were highly expressed in milk [39,40]. Interestingly, miRGalaxy discovered a much greater number of DE miRs and isomiRs with statistical significance (*p*-adj < 0.05) and |Log2FC| ≥ 1 in comparison to the original study [34]. For example, outputs from the two analyses of miR-200b-3p and isomiRs are shown in Table 2.

Despite the wide variety of output files that users can explore, we have made visible only the main result file in the Galaxy, which will bring a more user-friendly overview of the results. The visible outputs in the history menu are the MultiQC summarization of the quality check before and after the adapter trimming, count matrix tables, and isomiR profile report from IsoRead tool and DE report from miRViz tool. Advanced users may also access hidden outputs such as mapping files, isomiR database, and others from the history menu for a more in-depth view.

## 4. Conclusions

Here we present the miRGalaxy framework, an open-source, Galaxy-based platform for miR and isomiR analysis to address several essential needs in the field. We employed a set of different biofluid and liquid biopsy sRNA-seq data to demonstrate the usability of miRGalaxy, and generated a comprehensive picture of differentially expressed miRs and isomiR species. Despite recent exciting progress in the field of liquid-biopsy-based diagnostics, in-depth investigation of the biofluids miR-ome remains challenging. We have shown that the miRGalaxy framework can be successfully deployed for such datasets to identify, annotate, and discover miR/isomiR-ome and its behavior.

Since our tool suite is implemented in the Galaxy platform, users with less bioinformatics knowledge are able to perform elaborate workflows in a user-friendly and automated way. Moreover, due to the pipeline modular structure, it will also enable advanced users to customize the workflow to their needs. Further developments will include support for more reference genomes (e.g., species), and tool access points not only via the Toolshed installation and the stand-alone Docker flavor but also through various public Galaxy servers worldwide.

## Figures and Tables

**Table 1:** (**A**) The most abundant RefSeq miRs, template (t) and non-template (nont) isomiRs in all milk and plasma samples [34] detected by miRGalaxy. (**B**) The most abundant RefSeq miRs, template (t) and non-template (nont) isomiRs in all cancer (PDAC) and control (asymptomatic individuals) samples [35] detected by miRGalaxy.

A. miRs and isomiRs			
Plasma		Milk	
Name	Raw Counts	Name	Raw Counts
**RefSeq miRs**			
miR-486-5p_chr8(−)_chr8(+)	341,413	miR-148a-3p_chr7(−)	14,357,338
miR-122-5p_chr18(+)	190,578	miR-30a-5p_chr6(−)	1,753,796
miR-320a-3p_chr8(−)	66,951	miR-146b-5p_chr10(+)	915,663
miR-92a-3p_chr13(+)_chrX(−)	51,339	miR-26a-5p_chr12(−)_chr3(+)	667,270
miR-423-5p_chr17(+)	49,904	let-7f-5p_chr9(+)_chrX(−)	616,265
**Template isomiRs**			
miR-122-5p_chr18(+)_t_0_−1	201,561	miR-200a-3p_chr1(+)_t_0_−1	1,628,344
miR-486-5p_chr8(−)_chr8(+) _t_0_−1	177,874	miR-148a-3p_chr7(−)_t_0_−1	1,170,712
miR-423-5p_chr17(+)_t_0_−2	171,672	miR-146b-5p_chr10(+)_t_0_−1	620,583
miR-451a_chr17(−)_t_0_−1	163,779	miR-146b-5p_chr10(+)_t_0_+1	562,077
miR-486-5p_chr8(−)_chr8(+)_t_0_−2	75,436	miR-21-5p_chr17(+)_t_0_−1	558,349
**Non-template isomiRs**			
miR-486-5p_nont_0_+1_A	146,122	miR-148a-3p_chr7(−)_t_0_−1_ nont_0_+1_C	3,242,405
miR-486-5p_nont_0_+1_T	93,666	miR-148a-3p_chr7(−)_t_0_−1_ nont_0_+1_A	708,482
miR-122-5p_nont_0_+1_A	67,547	miR-148a-3p_chr7(−)_t_0_−1_ nont_0_+1_G	540,724
miR-320a-3p_chr8(−)_t_0_−1_ nont_0_+1_T	22,925	miR-200a-3p_chr1(+)_t_0_−2_ nont_0_+1_A	162,583
miR-122-5p_chr18(+)_t_0_−2_ nont_0_+1_C	19,211	miR-26a-5p_chr12(−)_chr3(+)_t_0_−1_nont_0_+1_C	153,539
**B. miRs and isomiRs**			
**Cancer (PDAC)**		**Control (Asymptomatic Individuals)**	
**Name**	**Raw Counts**	**Name**	**Raw Counts**
**RefSeq miRs**			
hsa-miR-26a-5p_chr12(−)_chr3(+)	6,005,560	hsa-miR-26a-5p_chr12(−)_chr3(+)	5,825,421
hsa-let-7f-5p_chr9(+)_chrX(−)	3,307,664	hsa-miR-191-5p_chr3(−)	3,375,427
hsa-miR-191-5p_chr3(−)	3,150,135	hsa-let-7a-5p_chr11(−)_chr9(+)_chr22(+)	3,227,687
hsa-let-7a-5p_chr11(−)_chr9(+)_chr22(+)	3,041,788	hsa-let-7f-5p_chr9(+)_chrX(−)	2,844,363
hsa-miR-22-3p_chr17(−)	2,616,893	hsa-miR-22-3p_chr17(−)	2,530,803
**Template isomiRs**			
hsa-miR-30d-5p_chr8(−)_t_0_+2	1,103,932	hsa-miR-30d-5p_chr8(−)_t_0_+2	1,167,321
hsa-miR-486-5p_chr8(−)_chr8(+)_t_0_−1	1,033,505	hsa-miR-486-5p_chr8(−)_chr8(+)_t_0_−1	1,114,672
hsa-miR-181a-5p_chr1(−)_chr9(+)_t_0_−1	866,979	hsa-miR-142-5p_chr17(−)_t_-2_−1	971,608
hsa-miR-21-5p_chr17(+)_t_0_+2	810,654	hsa-miR-181a-5p_chr1(−)_chr9(+)_t_0_−1	936,358
hsa-miR-142-5p_chr17(−)_t_-2_−1	808,717	hsa-miR-21-5p_chr17(+)_t_0_+2	718,611
**Non-template isomiRs**			
hsa-miR-92a-3p_nont_0_+2_AT	1,940,172	hsa-miR-92a-3p_nont_0_+2_AT	2,105,693
hsa-miR-486-5p_nont_0_+1_T	1,304,573	hsa-miR-486-5p_nont_0_+1_T	1,573,373
hsa-miR-92a-3p_nont_0_+2_AA	864,072	hsa-miR-92a-3p_nont_0_+2_AA	903,167
hsa-miR-486-5p_nont_0_+1_A	582,067	hsa-miR-486-5p_nont_0_+1_A	619,989
hsa-miR-92a-3p_nont_0_+2_TT	459,988	hsa-miR-92a-3p_nont_0_+2_TT	474,848

**Table 2:** Outputs of DE analyses of miR-200b-3p and isomiRs performed by miRGalaxy in comparison with the study of Rubio and co-workers [34] (only statistically significant results are shown).

miR and isomiR Names	Base Mean	Log2FC	*p*-adj
**Rubio and co-workers** [34]			
miR-200b-3p	22349.79	−7.74	1.67 × 10^−17^
**Output from miRGalaxy**			
miR-200b-3p_chr1(+)	2838.44	−9.29	1.15 × 10^−43^
miR-200b-3p_chr1(+)_t_+1_-1	80.73	−7.19	2.68 × 10^−13^
miR-200b-3p_chr1(+)_t_+1_+1	358.91	−8.26	2.18 × 10^−26^
miR-200b-3p_**chr1**(+)_t_+1_+1_nont_0_+1_A	693.30	−8.49	1.42 × 10^−25^
miR-200b-3p_chr1(+)_t_+1_+1_nont_0_+1_C	276.25	−8.16	4.46 × 10^−22^
miR-200b-3p_chr1(+)_t_+1_+1_nont_0_+1_T	1267.51	−7.70	4.16 × 10^−28^
miR-200b-3p_chr1(+)_t_+1_+2	101.54	−7.63	8.54 × 10^−18^
miR-200b-3p_chr1(+)_t_+1_0	92.47	−7.43	3.36 × 10^−15^
miR-200b-3p_chr1(+)_t_+1_0_nont_0_+1_T	56.09	−6.57	1.75 × 10^−09^
miR-200b-3p_chr1(+)_t_+2_+1_nont_0_+1_A	43.01	−6.41	2.49 × 10^−12^
miR-200b-3p_chr1(+)_t_+2_+1_nont_0_+1_T	106.81	−7.71	1.48 × 10^−18^
miR-200b-3p_chr1(+)_t_+2_+2	41.27	−6.40	2.34 × 10^−13^
miR-200b-3p_chr1(+)_t_0_-1	6656.60	−7.18	5.29 × 10^−25^
miR-200b-3p_chr1(+)_t_0_-1_nont_0_+1_C	32.03	−5.50	3.22 × 10^−09^
miR-200b-3p_chr1(+)_t_0_-1_nont_0_+1_G	118.22	−7.28	1.14 × 10^−14^
miR-200b-3p_chr1(+)_t_0_-1_nont_0_+1_T	129.70	−6.88	2.39 × 10^−17^
miR-200b-3p_chr1(+)_t_0_-1_nont_0_+2_GC	119.02	−6.26	3.31 × 10^−09^
miR-200b-3p_chr1(+)_t_0_-2	268.42	−7.65	1.51 × 10^−18^
miR-200b-3p_chr1(+)_t_0_-2_nont_0_+1_A	568.39	−8.70	8.14 × 10^−24^
miR-200b-3p_chr1(+)_t_0_-2_nont_0_+1_C	35.22	−5.96	2.94 × 10^−08^
miR-200b-3p_chr1(+)_t_0_-2_nont_0_+1_T	40.11	−6.22	4.41 × 10^−10^
miR-200b-3p_chr1(+)_t_0_+1	5005.92	−7.80	1.58 × 10^−32^
miR-200b-3p_chr1(+)_t_0_+2	150.83	−7.75	2.02 × 10^−20^
miR-200b-3p_nont_0_+1_A	260.75	−7.99	4.85 × 10^−19^
miR-200b-3p_nont_0_+1_G	33.11	−6.00	3.95 × 10^−10^
miR-200b-3p_nont_0_+1_T	861.69	−7.72	2.96 × 10^−15^
miR-200b-3p_nont_0_+2_CA	881.83	−8.24	6.25 × 10^−19^
miR-200b-3p_nont_0_+2_CC	331.61	−8.54	6.48 × 10^−23^
miR-200b-3p_nont_0_+2_CT	1424.88	−8.24	5.22 × 10^−26^

## Data Availability

Not applicable.

## Supplementary Materials

The following are available online at https://www.mdpi.com/article/10.3390/cancers13225663/s1, Table S1: Database files with all detected miRs and isomiRs (milk and plasma), Table S2: Count matrices (milk and plasma), Table S3: Differential expression results (milk and plasma), Table S4: Database files with all detected miRs and isomiRs (control and PDAC), Table S5: Count matrices (control and PDAC), Table S6: Differential expression results (control and PDAC), Figure S1: IsomiR profile report (milk and plasma), Figure S2: Differential expression of miRs and isomiRs report (milk and plasma), Figure S3: IsomiR profile report (control and PDAC), Figure S4: Differential expression of miRs and isomiRs report (control and PDAC).
