# Supplementary material for: miRGalaxy: Galaxy-Based Framework for Interactive Analysis of microRNA and isomiR Sequencing Data
Source: Cancers (Basel). 2021 Nov 12;13(22):5663. doi: 10.3390/cancers13225663 (PMC8616193; doi:10.3390/cancers13225663)

IsomiR profile report

sRNA length distribution

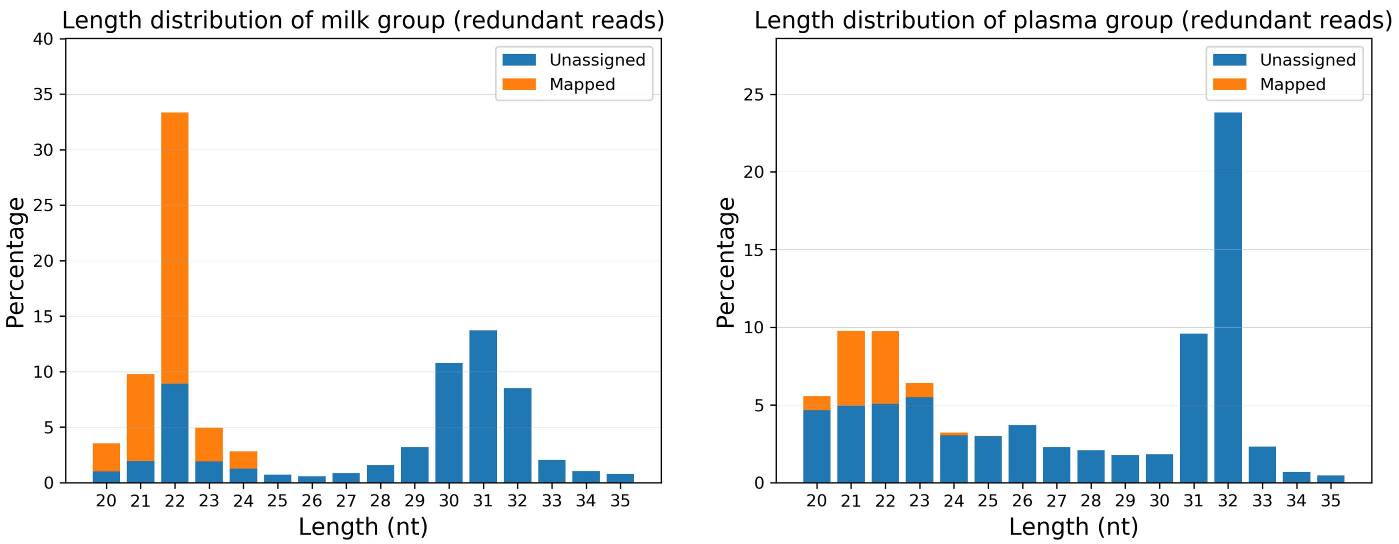

Mapped and unmapped reads to custom precursor arm reference DB (5p and 3p arms) in milk (left) and plasma (right) groups

Templated and non-templated isomiRs

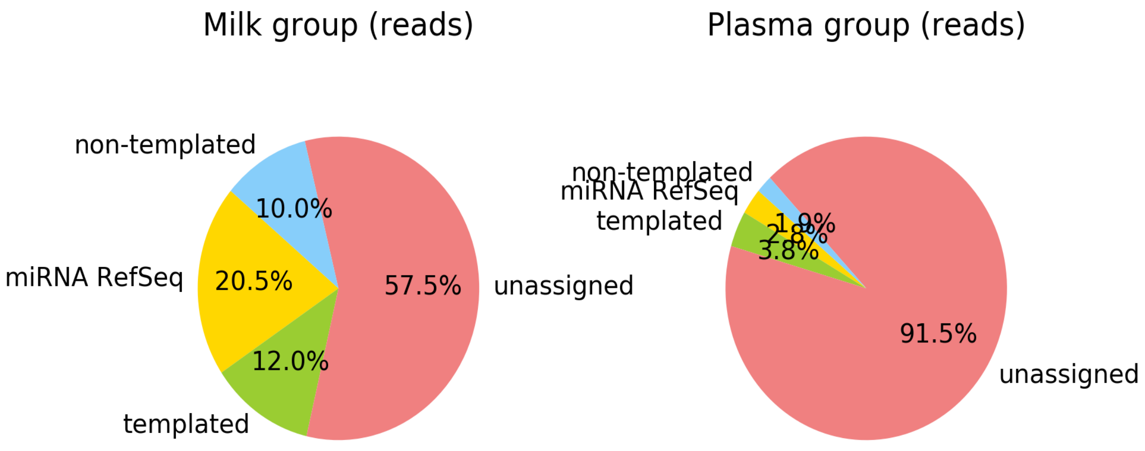

RefSeq miRNAs, templated isomiRs, non-templated isomiRs and unassigned sequences as percentage of total sRNA reads in milk (left) and plasma (right) groups

Templated isomiR subtypes

Templated isomiR profile (redundant reads)

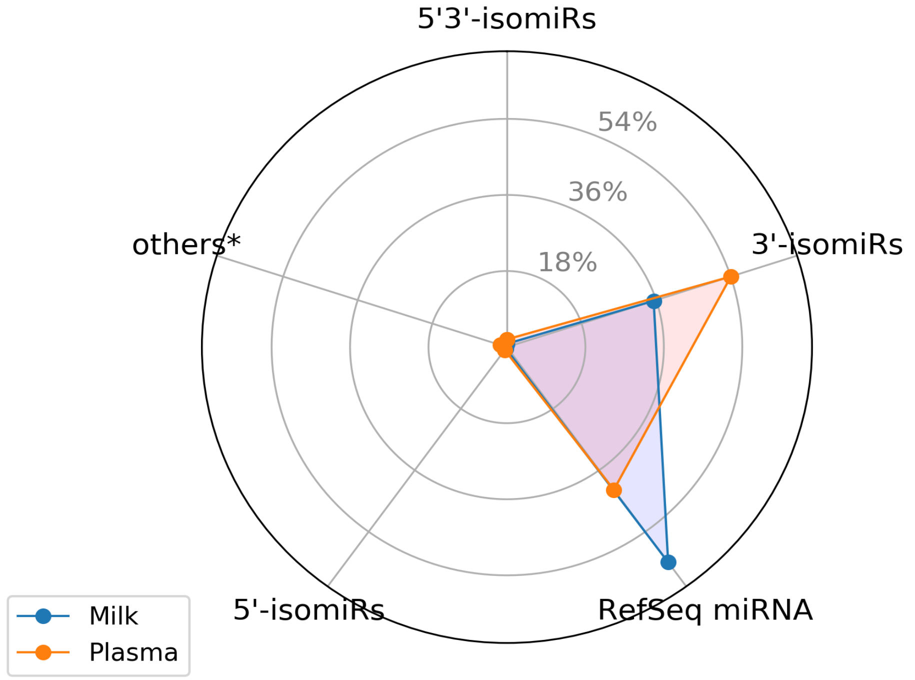

Templated isomiR profile (non-redundant reads)

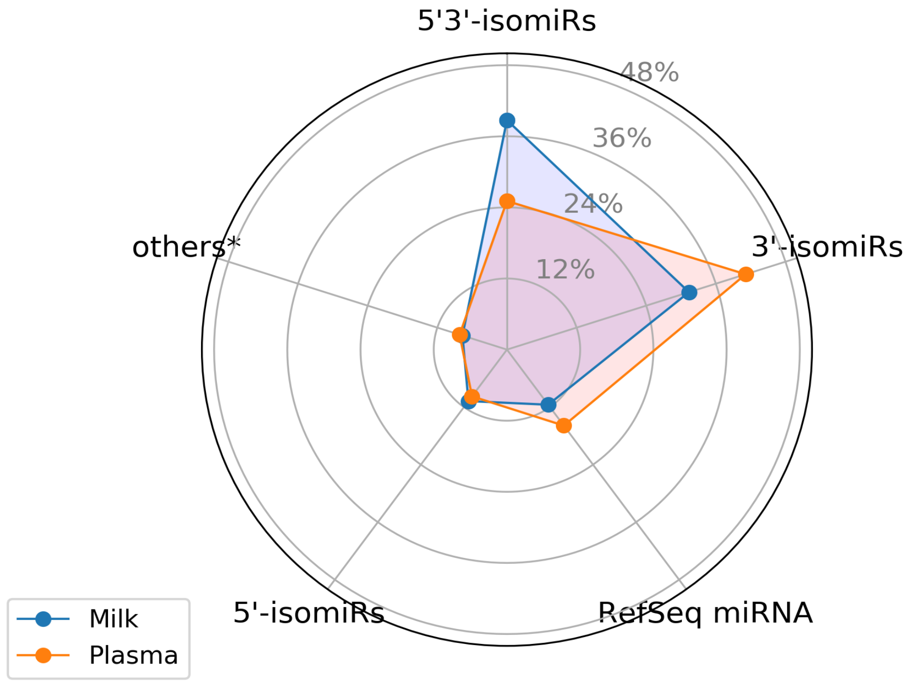

* IsomiRs potentially generated from multiple loci

Non-templated isomiRs

3'-end additions to RefSeq miRNAs and templated isomiRs

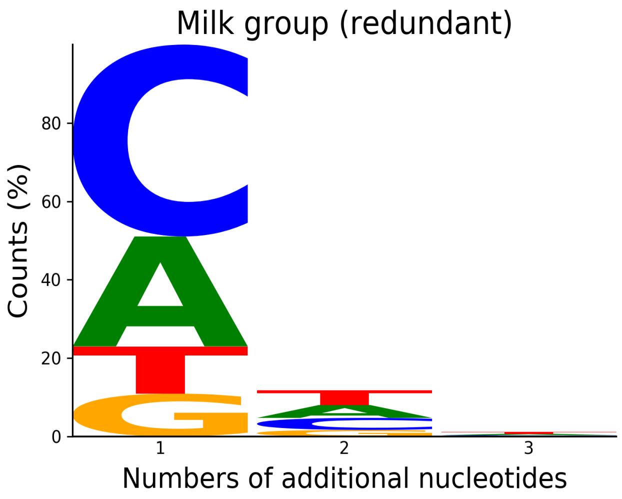

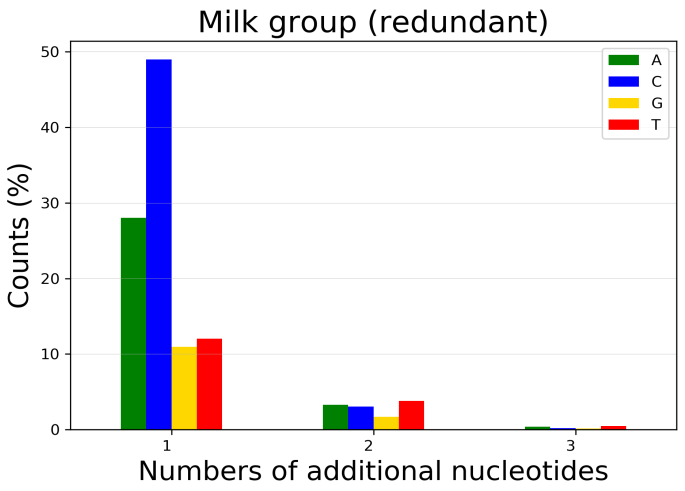

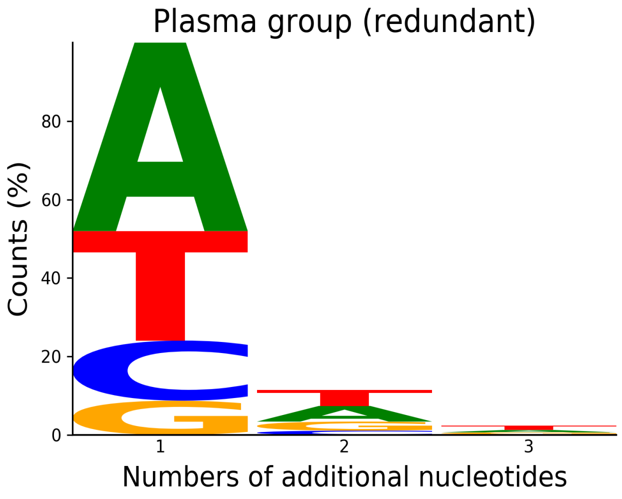

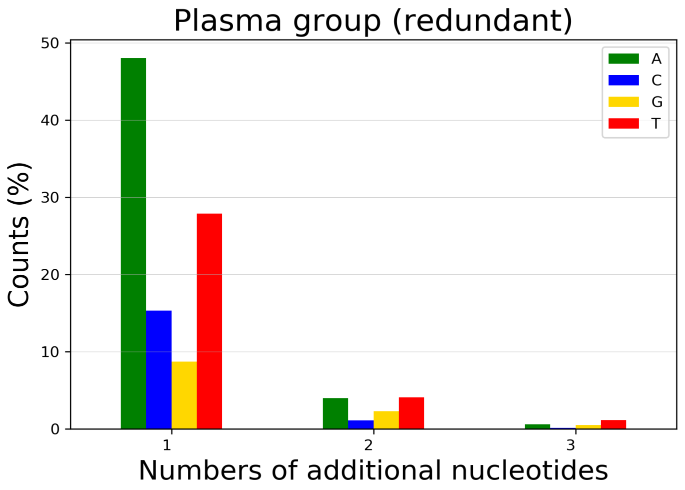

Supplement: Supplementary file 1 [file cancers-13-05663-s001.zip › Supplementary Data/Figure S1.pdf]
