# Supplementary material for: miRGalaxy: Galaxy-Based Framework for Interactive Analysis of microRNA and isomiR Sequencing Data
Source: Cancers (Basel). 2021 Nov 12;13(22):5663. doi: 10.3390/cancers13225663 (PMC8616193; doi:10.3390/cancers13225663)
Supplement: Supplementary file 1 [file cancers-13-05663-s001.zip › Supplementary Data/Figure S2.pdf]

Differential expression of miRNAs and isomiRs

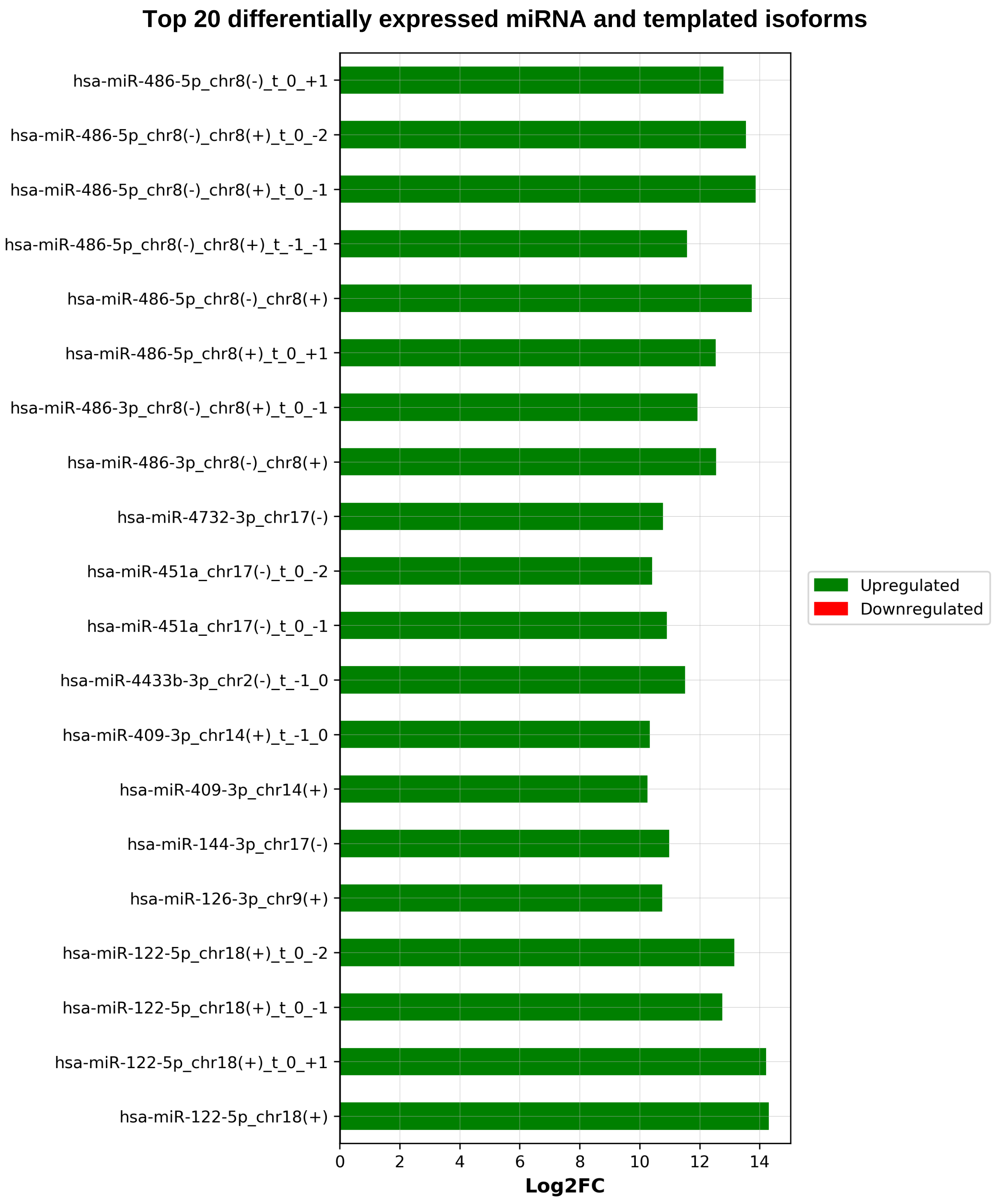

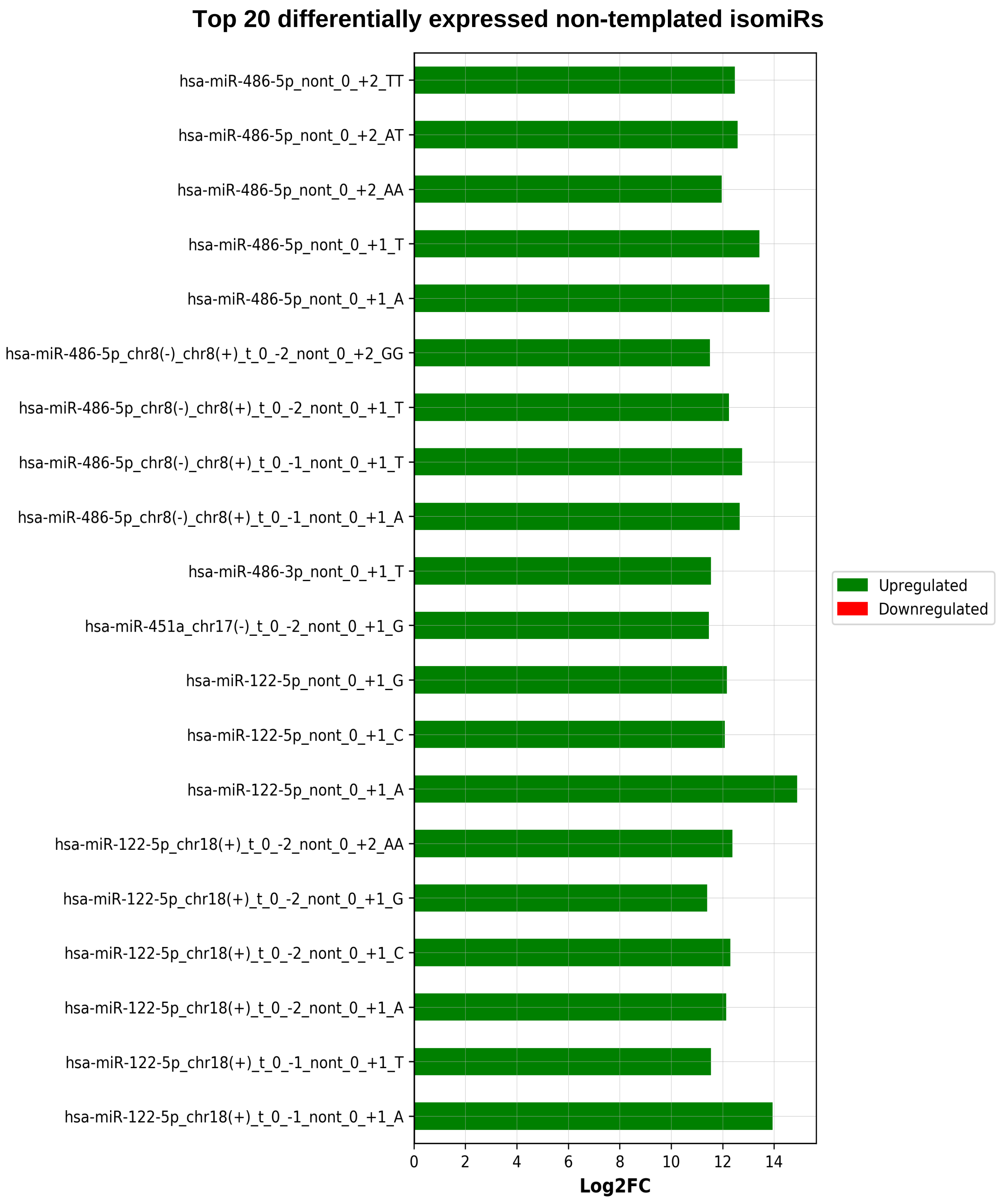
Top 20 differentially expressed non-templated isomiRs
hsa-miR-486-5p_nont_0_+2_TT
hsa-miR-486-5p_nont_0_+2_AT
hsa-miR-486-5p_nont_0_+2_AA
hsa-miR-486-5p_nont_0_+1_T
hsa-miR-486-5p_nont_0_+1_A
hsa-miR-486-5p_chr8(-)_chr8(+)_t_0_-2_nont_0_+2_GG
hsa-miR-486-5p_chr8(-)_chr8(+)_t_0_-2_nont_0_+1_T
hsa-miR-486-5p_chr8(-)_chr8(+)_t_0_-1_nont_0_+1_T
hsa-miR-486-5p_chr8(-)_chr8(+)_t_0_-1_nont_0_+1_A
hsa-miR-486-3p_nont_0_+1_T
hsa-miR-451a_chr17(-)_t_0_-2_nont_0_+1_G
hsa-miR-122-5p_nont_0_+1_G
hsa-miR-122-5p_nont_0_+1_C
hsa-miR-122-5p_nont_0_+1_A
hsa-miR-122-5p_chr18(+)_t_0_-2_nont_0_+2_AA
hsa-miR-122-5p_chr18(+)_t_0_-2_nont_0_+1_G
hsa-miR-122-5p_chr18(+)_t_0_-2_nont_0_+1_C
hsa-miR-122-5p_chr18(+)_t_0_-2_nont_0_+1_A
hsa-miR-122-5p_chr18(+)_t_0_-1_nont_0_+1_T
hsa-miR-122-5p_chr18(+)_t_0_-1_nont_0_+1_A
0
2
4
6
8
10
12
14
Log2FC
Upregulated
Downregulated

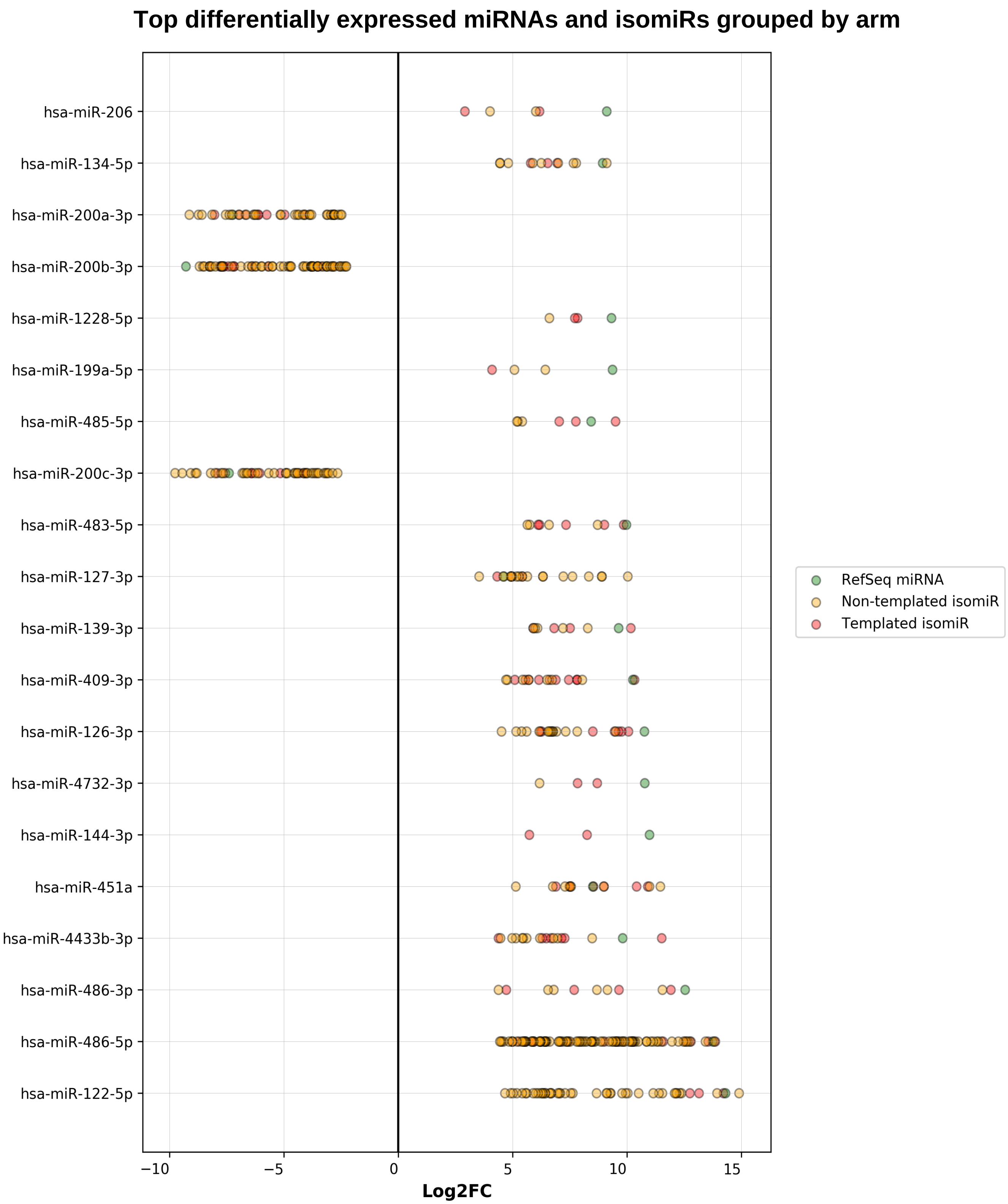

Top differentially expressed miRNAs and isomiRs grouped by arm
hsa-miR-206
hsa-miR-134-5p
hsa-miR-200a-3p
hsa-miR-200b-3p
hsa-miR-1228-5p
hsa-miR-199a-5p
hsa-miR-485-5p
hsa-miR-200c-3p
hsa-miR-483-5p
hsa-miR-127-3p
hsa-miR-139-3p
hsa-miR-409-3p
hsa-miR-126-3p
hsa-miR-4732-3p
hsa-miR-144-3p
hsa-miR-451a
hsa-miR-4433b-3p
hsa-miR-486-3p
hsa-miR-486-5p
hsa-miR-122-5p
−10
−5
0
5
10
15
Log2FC
RefSeq miRNA
Non-templated isomiR
Templated isomiR
