# Supplementary material for: miRGalaxy: Galaxy-Based Framework for Interactive Analysis of microRNA and isomiR Sequencing Data
Source: Cancers (Basel). 2021 Nov 12;13(22):5663. doi: 10.3390/cancers13225663 (PMC8616193; doi:10.3390/cancers13225663)
Supplement: Supplementary file 1 [file cancers-13-05663-s001.zip › Supplementary Data/Figure S4.pdf]

Differential expression of miRNAs and isomiRs

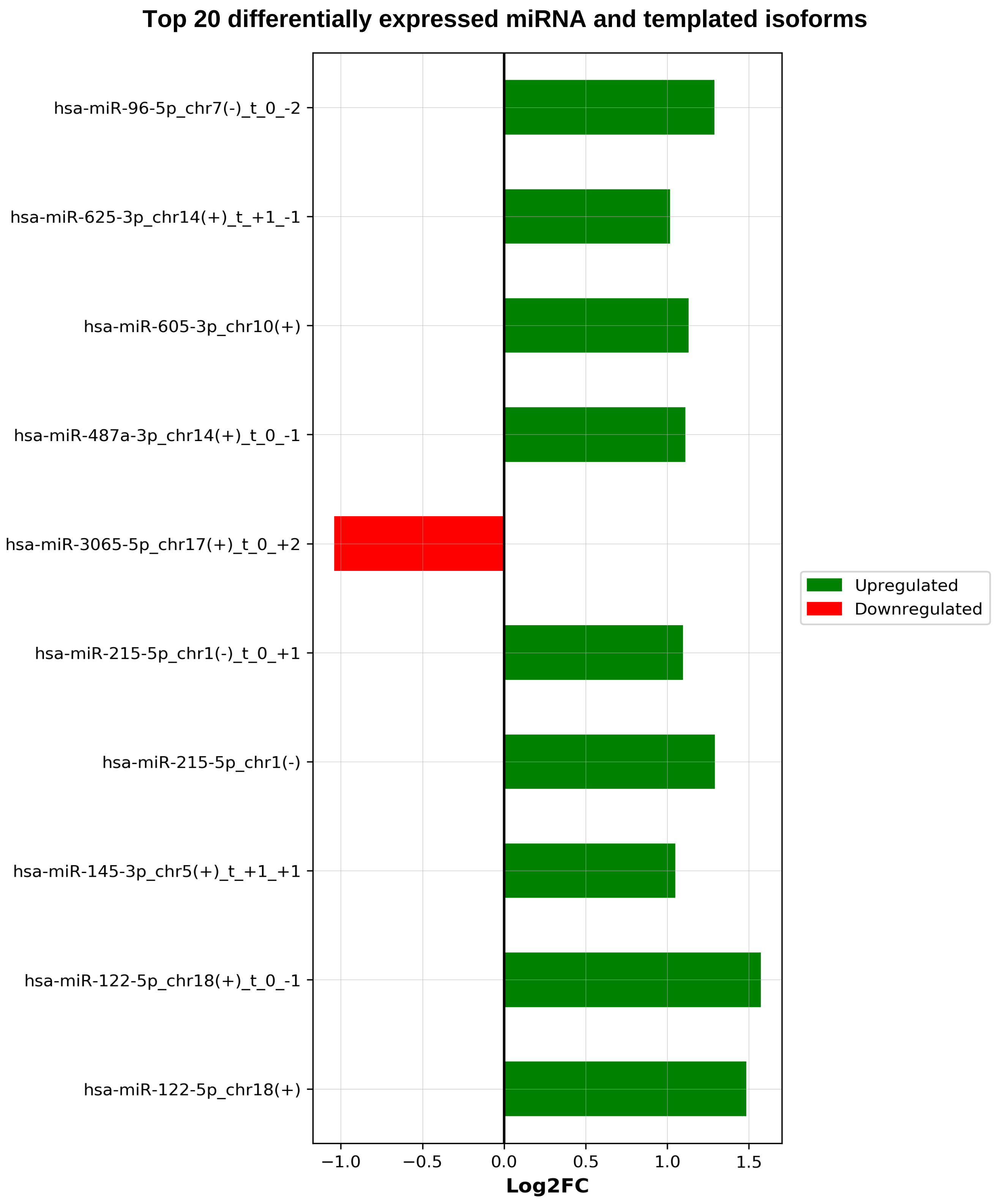

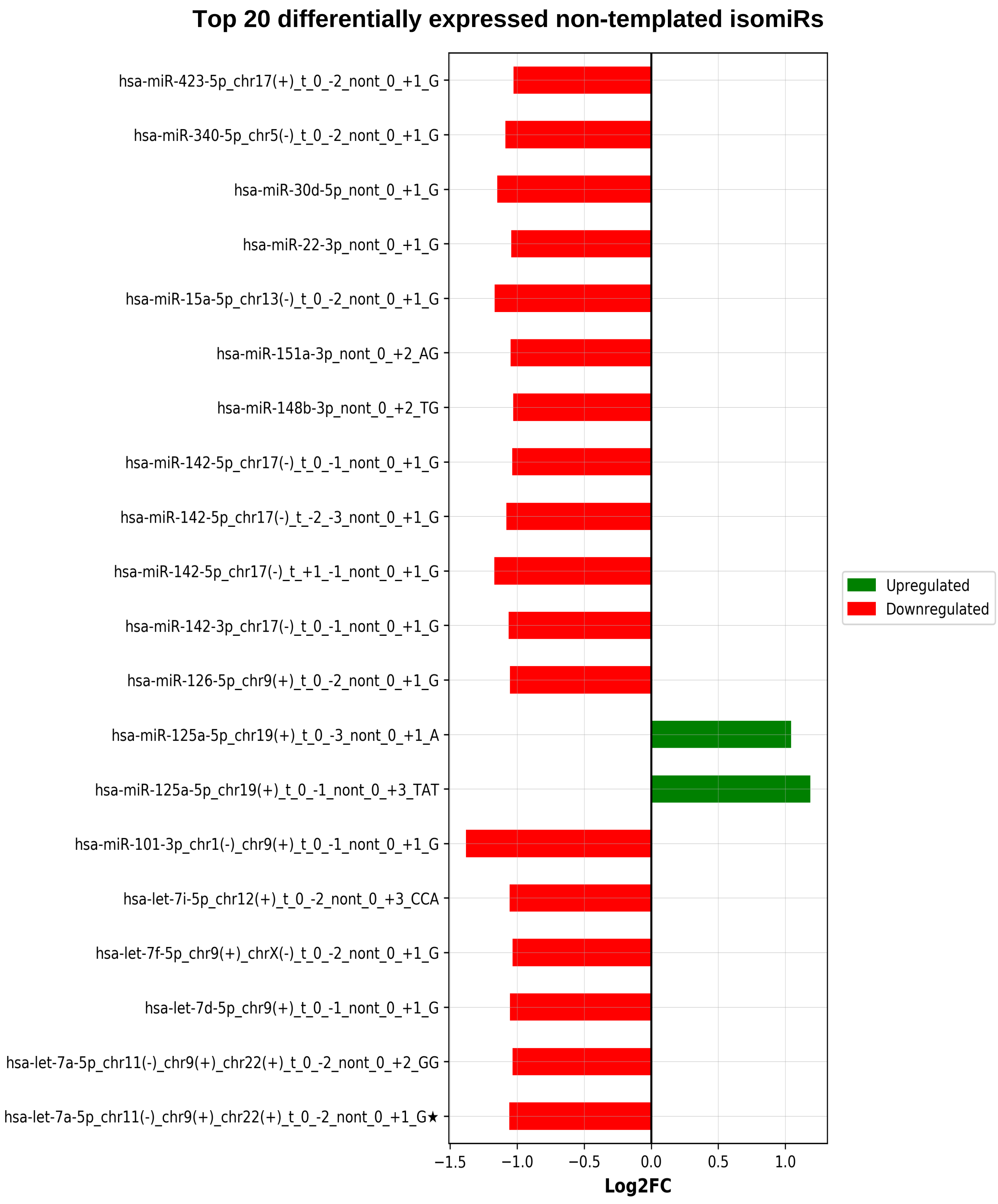

★ IsomiRs potentially generated from multiple loci

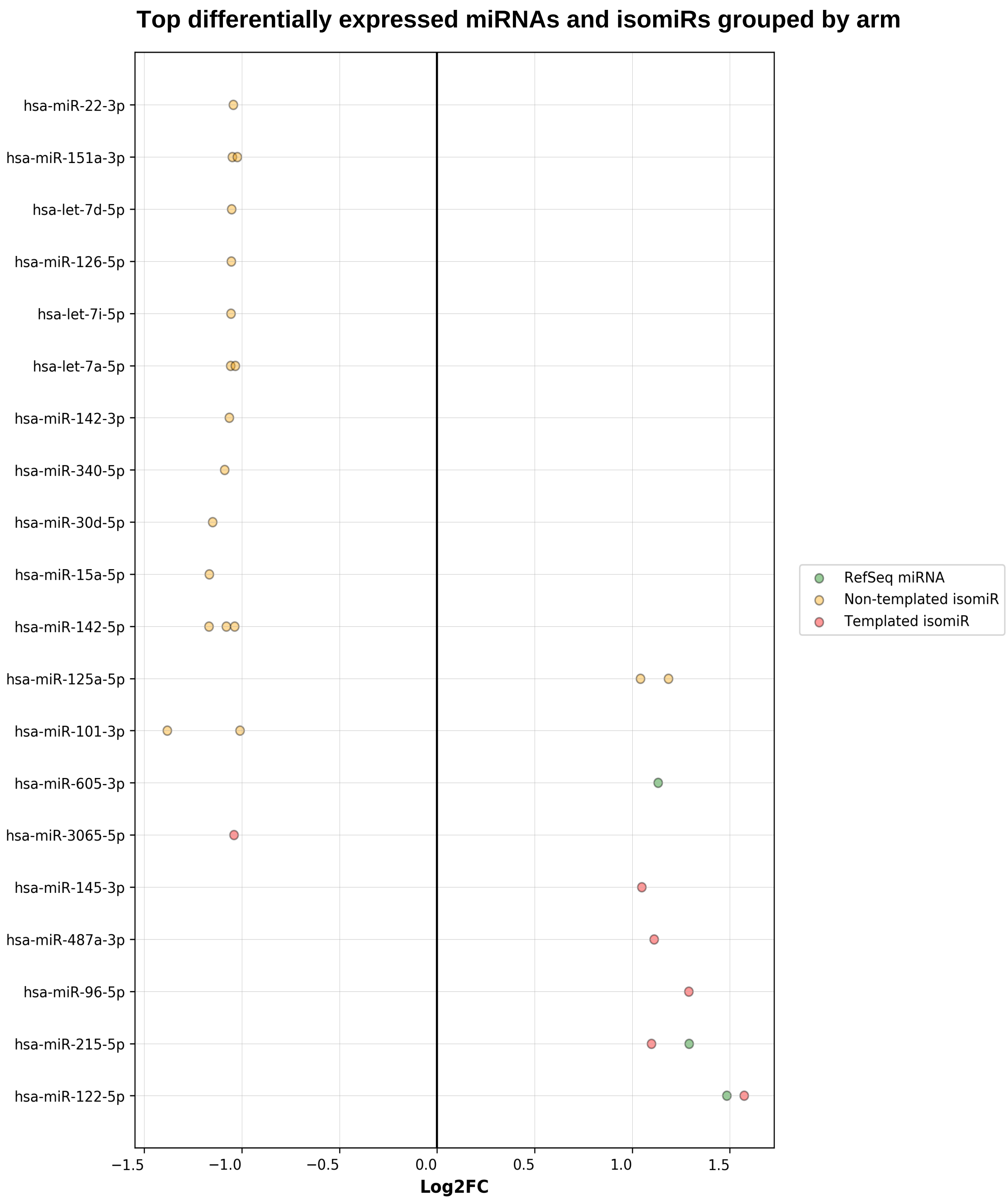
Top differentially expressed miRNAs and isomiRs grouped by arm
hsa-miR-22-3p
hsa-miR-151a-3p
hsa-let-7d-5p
hsa-miR-126-5p
hsa-let-7i-5p
hsa-let-7a-5p
hsa-miR-142-3p
hsa-miR-340-5p
hsa-miR-30d-5p
hsa-miR-15a-5p
hsa-miR-142-5p
hsa-miR-125a-5p
hsa-miR-101-3p
hsa-miR-605-3p
hsa-miR-3065-5p
hsa-miR-145-3p
hsa-miR-487a-3p
hsa-miR-96-5p
hsa-miR-215-5p
hsa-miR-122-5p
−1.5
−1.0
−0.5
0.0
0.5
1.0
1.5
Log2FC
RefSeq miRNA
Non-templated isomiR
Templated isomiR
